# Impact of Primary Immunodeficiency Diseases on the Life Experiences of Patients in Malaysia From the Caregivers’ Perspective: A Qualitative Study

**DOI:** 10.3389/fped.2022.846393

**Published:** 2022-03-31

**Authors:** Ruwaydah Ahmed Meelad, Intan Juliana Abd Hamid, Ilie Fadzilah Hashim, Zarina Thasneem Zainudeen, Firdaus Farhani Abu Bakar, Fahisham Taib, Norsarwany Mohamad, Ernest Mangantig, Intan Hakimah Ismail, Amir Hamzah Abdul Latiff, Lokman Mohd Noh

**Affiliations:** ^1^Primary Immunodeficiency Diseases Group, Department of Clinical Medicine, Institut Perubatan dan Pergigian Termaju, Universiti Sains Malaysia, Kepala Batas, Malaysia; ^2^Department of Paediatrics, School of Medical Sciences, Universiti Sains Malaysia, Kubang Kerian, Malaysia; ^3^Department of Paediatrics, Faculty of Medicine and Health Sciences, Universiti Putra Malaysia, Serdang, Malaysia; ^4^Pantai Hospital, Kuala Lumpur, Malaysia; ^5^Hospital Tunku Azizah, Kuala Lumpur, Malaysia

**Keywords:** Malaysia, primary immunodeficiency, inborn errors of immunity, thematic analysis, living experience, quality of life

## Abstract

**Introduction:**

Primary immunodeficiency diseases (PIDs) are chronic diseases that affect the various aspects of a patient’s life. However, the impact of living with PIDs is poorly described.

**Objective:**

This study aimed to explore the living experience challenges among the Malaysian caregivers of the patients with PID who underwent a follow-up in the Universiti Sains Malaysia or those registered members of the Malaysian PIDs Society.

**Methodology:**

The study was conducted from March 1 to May 30, 2021. The parents of children with PIDs were invited to participate for a semi-structured in-depth interview at the PID clinics in the USM. The estimated time of each interview was 30 min. The semi-structured interview was performed *via* a telephone call because of COVID-19 pandemic restrictions. The audio recording of each interview was transcribed and translated from Malay to English. Subsequently, a thematic analysis utilizing the ATLAS.ti software was performed.

**Results:**

The thematic analysis revealed five main themes, which are living with fear and anxiety with four sub-themes (sickness, psychological issues, fear of infections and hereditary issues), PID healthcare support struggles with four sub-themes (PID health system, treatment, diagnosis and financial issues), knowledge with two sub-themes (educational issues and disease understanding), social constraint with two sub-themes (relationships and social isolations) and coping with three sub-themes (acceptance, child health improvement and emotional hygiene).

**Conclusion:**

Living with fear and anxiety is a major theme impacting the living experiences of Malaysian patients with PIDs. Improvements in healthcare delivery and disease education are needed to ensure optimal quality of life.

## Introduction

Primary immunodeficiency diseases (PIDs) are inherited disorders caused by defects in the body’s immune system response and function, characterized by high susceptibility to opportunistic infection, autoimmunity, immune dysregulations and malignancies. In Malaysia, the frequency of PIDs is unknown because of the absence of a national patient registry for PID, and the estimated prevalence rate is 0.37 per 100,000 population (1). This number is considerably low compared with PID prevalence worldwide, which is 1:8,500–1:100,000 according to the PID registries from various countries (2). PIDs are chronic diseases that may require lifelong immunoglobulin replacement therapy (IRT). Malaysian healthcare is primarily administered by the Ministry of Health, with a two-tier healthcare system that includes both a government-based universal healthcare system and a co-existing private healthcare system. Most PID patients who receive intravenous IRT are subjected to hospitalization or admission into day care units for a minimum of 4–6 h. They require monthly admissions and this routine is required throughout their lives. Even though IRT prevents the exacerbation of infection, it has an enormous impact on a patient’s lifestyle and decision-making and may affect treatment compliance.

Qualitative thematic analysis has been used to explore challenges in caring for children with chronic diseases. For examples, patients with thalassemia are uncertain about their future, and this uncertainty is the major cause of their psychological stress (3). Many patients would have difficulty engaging in social events; however, most of them receive support from their spouses, families and friends. Patients’ challenges and worries should be carefully considered by healthcare practitioners, who should then competently initiate treatments and strategies by enabling patients to manage uncertainty with proper training and communication methods (3).

Children with PID have a wide range of coping mechanisms when dealing with distress, and these mechanisms include a positive mental attitude (4). Illnesses and treatment plans have a considerable impact on the family life, and the nature of these experiences greatly affects the daily lives of these patients (5). The established conceptual frameworks for describing the treatment burden were few and were mainly generated from the populations receiving other complex medical treatments rather than from index PID populations. Hence, we aimed to explore the experiences of patients with PIDs from the perspective of caregivers, focusing on the experiences of the caregivers and issues arising from the rearing of children with PID in Malaysia.

## Materials and Methods

### Study Site

The data of PID patients were collected from two centers. The Hospital Universiti Sains Malaysia (USM) is a major teaching and tertiary hospital located in the northeastern part of the Malaysian Peninsula. It is a 747-bedded hospital that covers many branches of pediatric services, including PID. The second hospital is the Advanced Medical and Dental Institution, which is a highly specialized research center located in northwest Malaysia. It is a 27-bedded hospital and actively provides services for patients with PID on an outpatient basis.

### Participants

The list of the caregivers of PID patients who had participated in a previous health-related quality-of-life (QOL) study was included (6). Patients with low QOL scores (mean < 67.26; defined as those parents or caregivers with a total score that is lower than the mean of the total score of the PID cohort studied as a comparison for cut-off points for low QOL scores) were identified and invited to participate in the present qualitative study (6).

This study was conducted through one-to-one interviews and audio recordings from March 1 to May 30, 2021. A semi-structured interview was conducted by a trained nurse who has no prior knowledge of the caregivers for 30 min. The interview questions were based on a previously published qualitative study (Table 1) (5). The questions were initially translated to the Malay language by a certified translator, and the interview was performed in the Malay language, given the fact that the majority of the participants were from Malay ethnicity. Consent was taken prior to the interview.

**TABLE 1:** List of questions asked in the semi-structured interview used in this study (5).

Questions
1. What is your child’s name? 2. How old is [child]? 3. Who lives here together?
4. Can you tell me a bit about what has happened in your family over the past couple of years? 5. What are the problems of caring for the PID* child? 6. How do the chronic conditions of a child with PID* influence your life? 7. How has communication been between you and your partner/your other children? 8. How do you and your family feel about the whole experience?
9. How is it for you to talk to me about this time in your life? 10. Is this something you have talked about much before? 11. How do you think what you have told me might be similar or different to what your partner would say?
12. What did you find helpful before/during/after transplant/IVIg*? 13. Can you also tell me about what you found unhelpful before/during/after transplant/IVIg*? 14. Have you noticed anything to have come from this experience?
15. How do you feel about your child who has been diagnosed with PID*? 16. How do you feel regarding caring for your child? 17. How do you feel about [child]’s health now?
18. What is your understanding of illness now? 19. What would you tell them about its cause, symptoms, treatment, and prognosis, inheritance?
20. How do you feel about the management of the healthcare system in your province?
21. How do you think the community causes suffering for your PID* child?

*IVIG, intravenous immunoglobulin, PID, primary immunodeficiency disease.

### Data Storage and Analysis

Owing to the physical restrictions imposed because of the COVID-19 pandemic, interviews were performed *via* a telephone call. Each interview was audio-recorded and transcribed verbatim for thematic analysis by the interviewer (FA) and was again translated to English by a certified translator. All the transcribed and translation materials were cross-examined by the principal investigator (IA) and two members of the research group (FA and IH) to ensure the validity of the content and that the meaning was not lost in translation. ATLAS.ti software version 9 was used. Immersion in the text and the identification of themes were performed according to the protocol of Braun and Clark (7). Themes and sub-themes were identified, and data triangulation was performed with the assistance of three authors (RAM, IH, and IH).

### Ethical Approval

The ethical approval for this study was obtained from the Human Research Ethics Committee of USM (USM/JEPeM/20040229).

## Results

### Demographic Characteristics

Thirteen caregivers were invited to participate in the interview. However, only 10 caregivers provided consent. The demographic characteristics of the caregivers (interviewees) and patients’ characteristics include those of a father and nine mothers (Table 2).

**TABLE 2:** Demographic characteristic of the PID caregivers respondent in this study.

Interviewee ID	Patients’ age (years)	Family members	Patient’s diagnosis	Status of interviewee
PNO* 1	26	4	Activated phosphoisonitide 3-kinase delta syndrome 2	Mother
PNO* 2	6	6	Severe combined Immunodeficiency	Mother
PNO* 3	17	7	Activated phosphoisonitide 3-kinase delta syndrome 2	Mother
PNO* 4	14	7	Hyper IgE syndrome	Mother
PNO* 5	11	6	XIAP defect	Mother
PNO* 6	19	3	X-linked chronic granulomatous disease	Mother
PNO* 7	13	6	Hyper-IgE syndrome	Father
PNO* 8	10	5	X-linked agammaglobulinemia	Mother
PNO* 9	41	7	Common variable immunodeficiency	Mother
PNO* 10	10	4	Hyper-IgM syndrome	Mother

*PNO, participant number.

### Thematic Analysis

**FIGURE 1:**
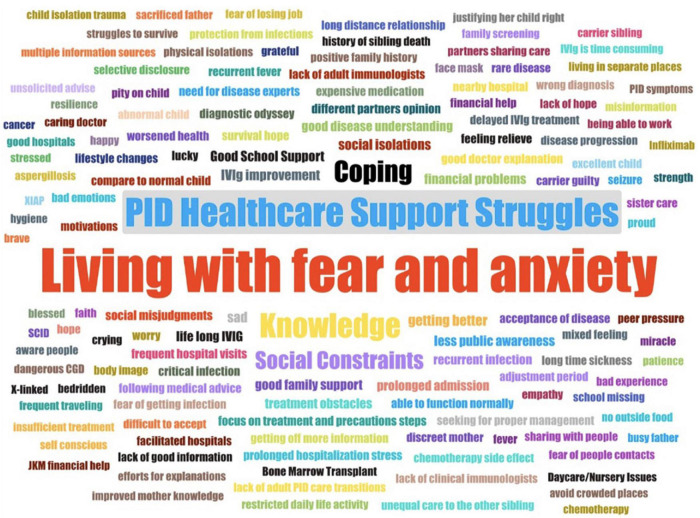
Wordcloud summarising all the themes and sub-themes identified through the thematic analysis of the interview transcripts.

The thematic analysis revealed five core themes, which showed that most parents had difficulty in caring for their children with PIDs. Their lives were affected in many ways, and lifestyle adjustment was required to accommodate the burden of treatment. The five themes identified were living with fear and anxiety, PID healthcare support struggles, knowledge, social constraint and coping (Table 3). The themes and sub-themes identified were summarized and illustrated using word clouds (Figure 1).

**TABLE 3:** Quotes, sub-themes and themes identified in this study through the analysis of interview transcripts.

Theme	Sub-theme	Examples of the quotes
Living with fear and anxiety	Sickness	…. (he had a lung infection, he was warded for 3 months and he was on antibiotic treatment.) (PNO 6) …. (he would always fall sick; he would always have a fever. And if he ever had fever or coughing, it would usually last around a month or so, he needs to see the doctor twice.) (PNO 9) …. (When she was 7, she was admitted in and out of the ward and had several operations for the lung infection that she had) (PNO 7)
	Psychological issues	……. (Quite sad. If I were not asked about it, I would feel just fine, but if I had to trace back the memories of it, it is quite emotional for me.) (PNO8) …… (We cannot be telling others how we feel inside) (PNO3)
	Fear of infection	……. (When we came back from work, we need to clean off everything) (PNO10) …… (But because we did not know about this, the thing needs to be much cleaner than it already is) (PNO2) …… (When we got to know this, we didn’t bring her out to public places, food-wise, we are really careful with that, so, whatever food that is exposed, we did not give her to eat, so it’s not so obvious after that. We kept her away from people who are sick. Fever or cough, we kept her away.) (PNO3)
	Hereditary issues	…. (if I am pregnant with a boy, I am afraid that I have that 50% chance of having another baby just like my son.) (PNO8) …. (So, when I wanted to get pregnant for the 4th time, my husband said, that he doesn’t want more, 3 is just enough.) (PNO 8) …. (Because my firstborn had passed and now my second one is sick, plus the doctor kept saying that my son wouldn’t live very long, it makes me feel that for as long as I live if I hadn’t had any child also I wouldn’t mind.) (PNO 9)
PID healthcare support struggles	PID health system	…. (After I came here and Dr. Intan had sent his blood to the United States for DNA tests, so now it’s clearer, and also, we knew that it is not NOD 2 but XIAP that had some connection with HLH and Crohn’s disease.) (PNO 5). …. (Yes. Now we are getting treatments in HKL, it used to be very hard and no place to go to seek treatment, but now, they have a specific ward just for them.) (PNO 9). …. (Before that I haven’t even met anyone from the immunologist team, they just gave me the name of the disease, the category. So then I looked it up. Then I got more confused than before, and I started to think of getting on ways of how to get him out from the hospital) (PNO10).
	Delay in the establishment of diagnosis	…. (I would like to stress on the journey of the diagnosis, and then the treatment and how trickle it is to get the proper treatment. So those are the parts that I need to stress out.) (PNO 10) …. (It is like she has one, but the doctor did not properly diagnose her yet.) (PNO 7) …. (He also took medication for TB, but it did not work because it is not TB…. I did not count those treatments as not helping but it’s just that at that point in time, the doctors had not found the real underlying cause of his problem.) (PNO 8).
	Treatment	…. (So, his life now depends on the IVIG.) (PNO8) …… (every time that we had to go for IVIG, we were placed in daycare, because the IVIG procedure would take about 6 h or so.) (PNO 10) …. (after this IVIG, there is no more coughing, and fever, he is like a normal child.) (PNO 5)
	Financial issues	…. (I would only have a financial problem because we are only gardeners.) (PNO 6) …. (No issue, he is still a child at that time, medication and treatment is still free, schooling also free as the admission is supported with a referral letter.) (PNO 9) …. (The most important thing is that our work are secured because we didn’t take a lot of leave) (PNO5)
	Child healthcare	…. (his father took turns looking after him in the wards because when he is admitted, it is not for a short period.) (PNO6). …. (I have a maid that is good at taking care of children.) (PNO 5)
Knowledge	Disease understanding	…. (Previously I only knew the type like XXX. When I entered MyPOPI, it was then I got to know that there are other types of PID that I haven’t even heard about.) (PNO 7). …. (But if we have the kind of society that lacks awareness and knowledge or we are surrounded with those kinds of people that does not have the awareness about this disease.)(PNO8)
	Educational issues	…. (he has been absent since last year.) (PNO 10) …. (At the age of 18, in March, he got his SPM results, and he got 7A’s…. he became a top student and one of the school prefects.) (PNO 6).
Social constraint	Social isolation	…. (We couldn’t send him to a nursery, because we are worried about him mixing around with other kids.) (PNO 7) …. (bit traumatized, we would only stay at home.) (PNO 2)
	Relationships	…. (But due to financial problems my husband went to Singapore and did not return since last year.) (PNO 6) …… (She is in Penang, and we are staying in Melaka.) (PNO 6) …. (I no longer have a husband.) (PNO4).
Coping	Acceptance	….(we need to accept the fate and be patient)(PNO3). …. (but when you see that there are other people’s children who are much sicker than our child, we would be thankful) (PNO1). …. (It feels normal, we just embraced and accepts it. No other feelings, we just accept what Allah gave to us.) (PNO 4).
	Emotional hygiene	……. (We just hope that his illness is in a stable state and doesn’t attack again.) (PNO5) …… (But yeah, the only thing that we can do right now is to find a way in making sure that he will get the best treatment, all the best ones, to secure his quality of life.) (PNO10) ……(My son was given until 18 years and with treatment and proper medication he might survive until 60 years) (PNO9).
	Child health improvement	…. (I can say that my son is as healthy as any other child.) (PNO 8) …. (So since he can walk again, he is working, he is a Grab driver.) (PNO9) …. (he applied to study teaching, and he got in.) (PNO6)
		

1. **Living with fear and anxiety**

Concerned about their children’s health, most parents expressed their experiences and fears of getting an infection and possessed overprotective behavior because of having a history of a long-time sickness, being subjected to hospitalization stress, worrying about inheritance and living with their emotional granting and suffering. This theme was further divided into four sub-themes:

i. **Sickness**

One of the major issues of PID patients is a long history of recurrent infection. It leads to parents’ time being spent in hospitals for their children’s treatment. Most of the parents experienced prolonged hospital stays for several months, especially at the beginning of sickness early in the childhood age. These experiences were expressed as extremely stressful times and perceived as bad encounters.

ii. **Psychological issues**

A sense of sadness, denial, community refusal, confusion and concerns regarding their children’s learning were expressed in the parents’ interviews. Some parents refused to talk about them but explained these difficult experiences, such as the sense of guilt of carrying an inherited disease, and some had concerns on their children’s future.

iii. **Fear of infection**

Owing to the low immunity of their children’s health, parents explained on their fear of contracting infections in the interview. Fortunately, some of the parents were acutely aware of precautionary measures, such as overcleansing and avoiding to eat in exposed places to avoid the transmission of infectious organisms.

iv. **Hereditary issues**

Parents raised concerns about the genetic inheritance in the family and expressed a concern that the disease might be passed down to future generations. Hence, some were against having children to avoid the transmissibility of life-threatening diseases to the offspring, and these acts seemed to be the mechanisms for evading the stressful circumstances that they were facing through.

2. **PID healthcare support struggles**

Parents were having difficulties in accessing standard treatment and diagnosis procedures and the inability to satisfy the financial needs for expensive treatment and daily supportive care for their children. This theme was divided into four main sub-themes, namely, PID health system, diagnosis, treatment and financial issues.

i. **PID health system**

The caregivers stated their good experiences with the current and available health facilities and medical services, such as bone marrow transplantation services. However, their preferences were related to clinical care provision by the tertiary hospitals and the experts in child-specific health (clinical immunology) to handle their needs. Some stated problems caused by the limited availability of clinical immunologists and local genetic testing centers.

ii. **Delay in the establishment of diagnosis**

Another important issue was related to the delay in establishing a PID diagnosis. Most parents struggled to arrive to the final disease diagnosis for their children, quoting a wrong diagnosis, time constraints, delayed timeline and the protracted process of investigations and diagnosis as a part of the reasons.

iii. **Treatment**

The journey that children with PIDs needed to undertake for appropriate care and treatment would represent another stress to parents and their children. The family has to endure life-long therapy, which all required full commitment from caregivers and patients. Immunoglobulin replacement therapy is considered one of the main lifelong therapies for patients with PIDs. It plays an important role in improving child health but is time consuming and requires planning with life adjustments.

iv. **Financial issues**

The major concern was related to the financial provision for the medical expenses of children, especially in the current “status quo” governmental support for adult patients with PIDs and their families. Some parents received financial help from various sources, and others were facing issues regarding job security, employment and support.

v. **Child healthcare**

The responsibility of raising and caring children is mainly shouldered by parents, especially mothers. Although filial care would allow family needs to be supported at hospitals and settings in our local context, this may be insufficient. Only some of the caregivers highlighted receiving help from their siblings, grandparents, extended family members and maid in child care.

3. **Knowledge**

This theme focuses on the experience when patients seek information regarding PID at school or in public domains. The two main sub-themes covered were disease understanding and educational issues.

i. **Disease understanding**

This section was related to the parental understanding of PID. They described the sources of their information and their inability to explain it to others and the understanding of PID among the close relatives. Our data suggested a vacuum of knowledge at the early onset of child sickness because of the rarity of the diseases. Nevertheless, their knowledge and awareness improved when they received support from medical teams. They multiplied their efforts to search PID information. All respondents reported facing the misjudgments and misunderstandings of their children’s health by people close to their families.

ii. **Educational issues**

Absenteeism in school mostly due to frequent hospital visits for immunoglobulin replacement treatment or multiple hospital admissions as a result of recurrent infections was the main concern for parents. Some of these children were very talented and showed excellent performance in their studies. Caregivers mentioned about an extended support from school teachers and healthcare providers when managing school issues and ensuring compliance to treatment schedules.

4. **Coping**

Some parents accepted their child’s diagnosis through positive reflection and modified their lives accordingly. They appeared to have strong spiritual faith and were grateful, stating their acceptance and surrender to God’s will. They described their children’s health improvement over time after regular treatments, such as intravenous immunoglobulin (IVIg) replacement therapy and BMT. This theme was divided into three main categories: acceptance, child health improvement and emotional hygiene.

i. **Acceptance**

Their long journey with a high sense of responsibility and acceptance to their current circumstances has led them to be more hopeful and faithful.

ii. **Child health improvement**

Most parents provided personal accounts of the severe infections of their children before the introduction of IVIg replacement therapy. They observed dramatic health improvement leading to minimal episodes of severe infections.

iii. **Emotional hygiene**

Some parents vented and shared their ill feelings and emotional burden. They felt encouraged to develop inner strength when facing daily struggles. They used self-motivation and perseverance to avoid being defeated by stress.

5. **Social constraints**

Most of the affected parents preferred home isolation and were against attending social events with friends and families. Furthermore, their relationships with their children and spouses have shown some gaps but were strengthened because of their children. Some families experienced parental separation or divorce, and some complained about lacking family time with their other children. This theme was divided into two main sub-themes: relationships and social isolation.

i. **Relationships**

Despite the fact that caregivers faced enormous challenges with their children’s health conditions, most of the parents showed strong relationships by sharing responsibility in taking care of their PID children. Some parents admittingly lived separately to earn higher incomes to support their children’s healthcare needs.

ii. **Social isolation**

Parents sacrificed their time, primarily focusing on their children’s benefit. They preferred to spend more time being at home rather than outdoor gatherings. Some children appeared to have problems in developing peer relationships as their parents did not allow them to mix with other children, indirectly wanting to avoid undesired infections.

## Discussion

The goal of this study was to explore the living experience and social issues related to the challenges faced by families caring for members with PIDs. To the best of our knowledge, this would be the first qualitative PID cohort study in Malaysia. Psychosocial themes were identified. The two main themes were identified through thematic analysis: living with fear and anxiety and PID support and struggles. The recurrence of ill feelings was observed in the extracted data, including the burden imposed by hospitalization and fear of the children’s illnesses and anguish for the inability to prevent the current state of life. This finding suggests that living and caring for patients with chronic and potentially fatal diseases are bad experiences (8).

The fear and worry of a child’s future health and concern that the hereditary disease might affect siblings in the family were identified problems. The negative stigma has indirectly affected the psychological health of children, and this effect was observed in children with thalassemia in Iran and Singapore (3, 9).

Parents recognized their struggles and protracted journeys to reach a definitive diagnosis, which would have delayed the diagnoses and indirectly affected their children’s chance for early bone marrow transplantation and immunoglobulin replacement therapy. The absence of pediatric immunologists leads to frequent hospital visits, which are time consuming and exhaustive. In addition, the journey to main tertiary hospitals is exhausting. Parents have to find additional jobs to financially cover their children’s expensive medications. The family budget may not be sufficient at the pursuit and maintenance of a healthy lifestyle, which includes the basic needs of the family, such as food and leisure (8). Governmental support is essential to the assurance of financial support to these families. The government currently subsidizes the cost of intravenous immunoglobulin replacement therapy for pediatric PID patients in Malaysia. However, the assistance might be expanded to include the cost of diagnostic testing, such as genetic tests.

Some information regarding PIDs are often misleading according to the caregivers. At the community level, many people misunderstand children’s health conditions, and the need for social support is often neglected. Other recurring issues were the misjudgments of others on the families (5). The awareness and inclusiveness of community education programs ensure that their needs are not excluded. Limited PID awareness among the healthcare professionals have been identified as one of the major challenges in Malaysia (10).

The parental knowledge and understanding of their children’s illnesses seem satisfactory. Parents were able to share and explain collectively their children’s medical conditions to other people. Parents who demonstrated high levels of medical knowledge often use “jargons” and attempted to find answers individually to their medical questions. This behavior has been reported in other studies (3, 8).

School absenteeism has raised concerns about the negative effects of PIDs on children. Missing social events and public gatherings, as a result of families living in isolation, reflects the great sense of parental responsibility on the need for modifications and abdications for children with PIDs (8, 11). The lack of support can lead to the physical and psychological exhaustion of caregivers, in addition to social isolation (8).

To some extent, parents had a stable relationship and even siblings showed strong bonds with their patients. A study investigating PID parents’ experience after a bone marrow transplant have identified gender difference as a dominant factor affecting the care of their children and also conflicts in their relationships and lives (5). Filial care was commonly provided by the spouse and extended family (8).

Parents coped by having spiritual sense, hope and perseverance. The strategies were getting a strong and persistent response, self-distraction and comparing their own position with those of unfortunate families with severe illnesses (3). One of the helpful coping strategies was assuming that their children’s health improvement is similar to that of other children. They would try curative treatments, such as bone marrow transplant, to increase their hope regarding their children’s survival, and hence, they were hoping for a “normal” life (3, 5).

Although the possible limitations of our study were recognized, most of the themes in our analysis were corroborated by similar known themes from earlier investigations. We believe that this is universal even when a small number of caregivers are involved in our study. We have reached a point of saturation whilst probing and exploring the qualitative data. This study was conducted in Malay language, and hence, the analysis may have lost the real meaning of the data during direct translation. The interview, which was done *via* a telephone call, may have weakened the data because the non-verbal cues during the interview sessions were not interpreted. Many attempts have been performed to combat these issues, including the cross-checking of all materials transcribed, translated data by the principal investigators and triangulation of data analysis. Due to the small sample size, it was not possible to divide the interview feedback analysis into different PID categories. Future studies involving multiple centers or countries may be able to solve this problem.

## Conclusion

Living with PID is not easy for families or children. It is a time-consuming experience in all aspects of life (social, emotional and even financial aspects), and it has an enormous impact on children’s future learning and health. A holistic approach is required from diagnostics, treatment and long-term planning including the essential presence of clinical immunologists in the Malaysian healthcare system.

## Data Availability Statement

The original contributions presented in the study are included in the article/supplementary material, further inquiries can be directed to the corresponding author/s.

## Ethics Statement

The studies involving human participants were reviewed and approved by the Human Research Ethics Committee Universiti Sais Malaysia (USM/JEPeM/20040229). The patients/participants provided their written informed consent to participate in this study.

## Author Contributions

RAM: conceptualization, methodology, validation, formal analysis, investigation, resources, data curation, writing—original draft, and writing—review and editing. IA: conceptualization, methodology, validation, formal analysis, investigation, resources, data curation, writing—original draft, writing—review and editing, supervision, and project administration. IH and ZZ: conceptualization, methodology, validation, formal analysis, and writing—review and editing. FA: methodology, validation, formal analysis, investigation, resources, data curation, writing—review and editing, and project administration. FT: methodology, validation, investigation, resources, data curation, writing—review and editing, and physician in charge of patients. NM, II, AA, and LM: conceptualization, writing—review and editing, and physician in charge of patients. EM: conceptualization, methodology, formal analysis, data curation, writing—review and editing. All authors contributed to the article and approved the submitted version.

## Conflict of Interest

The authors declare that the research was conducted in the absence of any commercial or financial relationships that could be construed as a potential conflict of interest.

## Publisher’s Note

All claims expressed in this article are solely those of the authors and do not necessarily represent those of their affiliated organizations, or those of the publisher, the editors and the reviewers. Any product that may be evaluated in this article, or claim that may be made by its manufacturer, is not guaranteed or endorsed by the publisher.
